# Supplementary material for: Structural Quality of Services and Use of Family Planning Services in Primary Health Care Facilities in Ethiopia. How Do Public and Private Facilities Compare?
Source: Int J Environ Res Public Health. 2020 Jun 12;17(12):4201. doi: 10.3390/ijerph17124201 (PMC7345433; doi:10.3390/ijerph17124201)
Supplement: Supplementary file 1 [file ijerph-17-04201-s001.pdf]

Supplementary Materials

Table S1. Description of variables used in the analysis.

Variable	Type of variable and/or definitions
**ESPA+ 2014 DATASET**	
**Facility type and location**	
Facility type	Binary (public, private); public included government and military facilities (health post, health centre, and primary hospital), whereas private included private for profit, private for-not-profit (lower clinic, medium clinic, higher clinic, and primary hospital)
Urban/rural location	Binary (urban, rural), classified based on the whether the facility is located in urban or rural areas
Region	Categorical, 11 administrative regions
**Facility's structural aspect**	
**Material resources**	
**Basic amenities/infrastructure**	
Availability of electricity/generator	Binary (yes, no); whether the health facility possessed electricity/generator
Availability of functional landline telephone	Binary (yes, no); whether the health facility possessed functional telephone/cell phone
Availability of cell phone	Binary (yes, no); whether the health facility possessed functional cell phone
Availability of functional computer	Binary (yes, no); whether the health facility possessed functional computer
Access to email for two hours in a day	Binary (yes, no); whether the health facility get email for two hours/day
Access to water supply	Binary (yes, no); whether the health facility possessed water supply
**Equipment and supplies**	
Availability of body weight measurement tool	Binary (yes, no); whether the facility showed functioning weight scales.
Availability of Blood Pressure (BP) measurement tool	Binary (yes, no); whether the facility showed functioning BP measurement apparatus.
Availability of stethoscope	Binary (yes, no); whether the facility showed functioning stethoscope
Availability of examination light	Binary (yes, no); whether the facility showed functioning examination light
Availability of exam couch	Binary (yes, no); whether the facility showed examination couch for examining clients
Availability of sample FP methods	Binary (yes, no); whether the facility showed sample FP methods for counselling
Availability of visual aid and leaflet	Binary (yes, no); whether the facility showed visual aid and leaflet for demonstration during counselling
Availability of pelvic model for demonstrating IUD use demonstration	Binary (yes, no); whether the facility showed pelvic model for demonstrating IUD use
Availability of model for demonstrating condom use	Binary (yes, no); whether the facility showed model for demonstrating condom

Mean number of contraceptive methods offered and/or prescribed	Continuous (0-12); the mean number of contraceptive methods offered or prescribed in the facility. The contraceptive methods included combined oral contraceptive pills, progestin only pills, progestin only injectables, male condom, female condom, implant, Intrauterine Device (IUD), periodic abstinence, emergency pills, female sterilization, and vasectomy (male sterilization), and lactational amenorrhea.
Mean number of infection prevention precaution measures	Continuous (0-14); the mean number of infection prevention precaution measures involved in the facility. It included availability of running water, handwashing soap, alcohol-based hand rub, waste receptacle, safety box, disposable latex glove, disinfectant/antiseptics, syringe, medical masks, gowns, eye protection goggle, standard precaution guidelines, and boots.
**Human resources and organisational structure, and provision of other reproductive and child health services**	
Health availability of twenty-four hours/seven days	Binary (yes, no); whether facilities have health providers available to provide services for twenty-four hours of day (day, night, and week shifts)
Trained provider availability	Binary (yes, no); whether the provider received FP related training in the past 24 months before the survey
**Organisational structure**	
Quality assurance system	Binary (yes, no); whether or not the facility routinely carry out periodic audit of registers to see facility-wide review of clients/patients data
FP guidelines/protocols	Binary (yes, no); whether the facility possessed FP guidelines/protocols on the date of survey
Client chart/record	Binary (yes, no); whether the facility possessed client chart or recoding for taking notes about the clients during history taking and physical assessment
Supervision in the past six months	Binary (yes, no); Whether the facility received a supervisory visit from district/regional/ zonal/federal offices in the six months before the survey
Private room for providing counselling services	Binary (yes, no); whether the facility's possessed private room for counselling during FP services
Presence of user fee for FP services	Binary (yes, no); whether the facility collected users' fee for FP services.
Mean number of days/week that FP was provided	Continuous; mean number of days that FP services were provided in a week
**Provision of other reproductive and child health services**	
Antenatal care services	Binary (yes, no); whether the facility provided antenatal care services during the survey
Normal delivery services	Binary (yes, no); whether the facility provided normal delivery services during the survey
Services for under-five children	Binary (yes, no); whether the facility provided under five services during the survey
Services for the prevention of mother-to-child transmission (PMTCT) of HIV	Binary (yes, no); whether the facility provided HIV test services during the survey
Diagnosis and treatment STI	Binary (yes, no); whether the facility provided diagnosis and treatment STI services during the survey
**EDHS 2016 DATASET**	
**Sources of FP method by facility types**	Categorical; public (government health station/centre, government health post, and public pharmacy, and other public sector) , private (private clinics, pharmacy, non-governmental organisation's health facilities, private clinics, and private pharmacies, and other private medical)
**Characteristics of women**	
Age category in years	Categorical; 15-24, 25-34, 35+
Marital status	Binary (yes, no); currently married (married or in sexual union), currently unmarried (never married, divorced, widowed, separated)

Place of residence	Binary (yes, no); Urban, rural
Region	Categorical; 11 administrative regions
Religion	Categorical; Orthodox, Muslim, Protestant, others
Highest educational status	Categorical; none, primary, secondary+
Highest partner educational status	Categorical; none, primary, secondary+
Working/occupational status	Categorical; Not working (not employed/not working), working (*women who describe themselves as employed or engaged in a work that paid them in cash or in kind*)
Wealth index	Categorical; poor, middle, rich
Number of living children	Categorical; 0, 1-2,3-4,5+
Exposure to FP media	Binary (yes, no); whether a woman heard or read about FP methods in radio/television/newspaper/mobile message
Women decision making	Binary (yes, no); whether a woman able to make decisions alone or with her partner regarding about her health care, large household purchase, and visiting her families or relatives

FP- Family planning, BP- Blood Pressure, IUD- Intrauterine Contraceptive Device, STI- Sexual transmitted Infections, HIV- Human Immunodeficiency Virus.

Table S2. Facility's location, structure, and provisions of reproductive and child health services in PHCU facilities in Ethiopia, ESPA+ 2014 (n=1,094).

Variables	Private (n=139)		Public (n=955)	
	Frequency	Percent	Frequency	Percent
Facility's location and region				
**Urban/rural location**				
Rural	65	46.6	889	93.1
Urban	74	53.4	66	6.9
Material resources				
**Basic amenities**				
Availability of functional landline telephone	20	14.1	40	4.2
Availability of functional cell phone	84	60.6	140	14.6
Availability of functional computer	16	11.4	93	9.7
Availability of short-wave radio	1	0.7	0	0.0
Access to email at least two hours on a day	7	5.3	8	0.9
Access to water supply	126	90.6	614	64.3
Availability Electricity supply/generator	66	47.7	354	37.1
At least one basic amenity	134	97.7	761	79.8
**Equipment and supplies**				
Availability of body weight measurement tool	16	11.9	97	10.1
Availability of blood pressure measurement tool	19	14.0	96	10.0
Availability of stethoscope	133	95.8	678	71.0
Availability of examination light	114	81.9	340	35.7
Availability of exam couch	134	96.4	560	58.7
Availability of sample FP methods	75	54.2	427	44.7
Availability of visual aid and leaflet	75	53.8	507	53.1
Mean number of types of contraceptive methods in the facility	6(SD=2.4)		5(SD=2.1)	
Mean number of infection prevention precaution measures	8.2(SD=2.2)		6.8(2.6)	

FP-Family Planning. SD- Standard Deviation.

Table S2. Cont.

Variables	Private (n=139)		Public (n=955)	
	Frequency	Percent	Frequency	Percent
Human resources				
Health provider availability of twenty-four hours/seven days a week	22	15.7	206	21.6
Trained provider availability	46	33.5	613	64.2
Organisational structure				
System to collect client opinion	2	1.7	129	13.1
FP guidelines/protocols	40	28.9	465	48.7
Client chart/record to document client's clinical data	44	31.6	632	66.2
Supervision in the past six months	70	51.4	527	58.0
Private room for providing counselling services	123	88.6	783	82.0
Presence of user fee for FP services	109	91.6	3	1.5
Mean number of days/week that FP was provided	6.4 (SD=0.71)		5.1(SD=1.27)	
Provisions of other reproductive and child health services				
Antenatal care services	78	55.9	909	95.3
Normal delivery services	36	26.1	538	56.4
Service for under five children	123	88.5	933	97.7
Services for the prevention of mother-to-child transmission of HIV	15	10.7	179	18.7
Diagnosis and treat for STI	123	88.4	341	35.8

FP-Family Planning. STI- Sexual Transmitted Infections. HIV- Human Immunodeficiency Virus. SD- Standard Deviation.
